# Validity and reliability of data collected by community health workers in rural and peri-urban contexts in Kenya

**DOI:** 10.1186/1472-6963-14-S1-S5

**Published:** 2014-05-12

**Authors:** Careena Flora Otieno-Odawa, Dan Owino Kaseje

**Affiliations:** 1 Tropical Institute Of Community Health, Great Lakes University Of Kisumu, Miwani Road, Kisumu, Kenya

**Keywords:** validity, reliability, data, community health workers, rural, peri-urban, validité, fiabilité, données, agents de santé communautaire, rural, périurbain

## Abstract

**Background:**

Reliability and validity of measurements are important for the interpretation and generalisation of research findings. Valid, reliable and comparable measures of health status of individuals are critical components of the evidence base for health policy. The need for sound information is especially urgent in the case of emerging diseases and other acute health threats, where rapid awareness, investigation and response can save lives and prevent broader national outbreaks and even global pandemics.

Several successfully implemented health interventions have involved community health workers (CHWs) in reaching out to the community, and the Community Health Strategy is one such an intervention. The government of Kenya, through the Ministry of Public Health and Sanitation has rolled out the strategy as a way of improving health care at the household level. It involves CHWs collecting health status data at the household level, which is presented at community meetings in which the community discusses the results, identifies action areas, and plans activities for improving their health status.

**Methods:**

Ten percent of all households visited by CHWs for data collection in different sites (rural and peri-urban) were systematically selected and visited a second time by technically trained research team members. The test-retest method was applied to establish reliability. The Kappa score was used to measure reliability, while sensitivity, specificity, and positive predictive values were used to measure validity.

**Results:**

Inter-observer agreement between the two sets of data in both sites was good; most indicators measured slight agreement. However, some indicators demonstrated greater discrepancies between the two data sets (e.g. measles immunization). Specificity measures were more stable in Butere (rural), which had more than 90% in all the indicators tested, compared to Nyalenda (peri-urban), which fluctuated between 50% and 90%. There were variable reliability results in the peri-urban site for the indicators measured, while the rural site presented more stable results. This is also depicted in the validity measures in both sites.

**Conclusions:**

The paper concludes that there are convincing results that CHWs can accurately and reliably collect certain types of community data which has cost-saving implications, especially for resource poor settings.

## Abstract (fr)

**Résumé:**

**Contexte**

La validité et la fiabilité des mesures sont importantes pour l’interprétation et la généralisation des résultats de recherche. Des mesures valables, fiables et comparables de l’état de santé des individus sont une partie importante de la base de données probantes pour les politiques en matière de santé. Le besoin d’information fiable est particulièrement criant dans le cas des maladies émergentes et d’autres risques sanitaires graves, où une prise de conscience, une enquête et une intervention rapides peuvent sauver des vies et prévenir les épidémies nationales et même des pandémies mondiales.

Plusieurs interventions en santé réussies ont comporté un déploiement d’agents de santé communautaire (ASC) dans les collectivités; la stratégie en santé communautaire est l’une de ces interventions. Le gouvernement du Kenya, par le truchement du ministère de la Santé publique et de la Salubrité, a mis en oeuvre cette stratégie dans le but d’améliorer les soins de santé pour les ménages. Pour ce faire, il est nécessaire que les ASC recueillent des données sur l’état de santé des ménages, qui sont alors présentées dans le cadre de rencontres communautaires où l’on discute des résultats, détermine des champs d’action et planifie des actions afin d’améliorer leur état de santé.

**Méthodes**

Dix pour cent de tous les ménages visités par les ASC pour la cueillette de données dans différents milieux (ruraux et périurbains) ont été systématiquement rencontrés une deuxième fois par des membres de l’équipe de recherche ayant reçu une formation technique. La méthode du test-retest a été appliquée pour établir la fiabilité. L’indice Kappa a été utilisé pour mesurer la fiabilité, alors que la sensibilité, la précision et la valeur prédictive positive ont été calculées pour mesurer la validité.

L’accord entre les données des deux ensembles d’observateurs dans les deux milieux était bon, la plupart des indicateurs possédaient un accord faible. Toutefois, certains indicateurs montraient de plus grands écarts entre les deux ensembles de données (p. ex., la vaccination contre la rougeole). Les mesures de spécificité étaient plus stables dans le district de Butere (milieu rural) qui a obtenu plus de 90 % dans tous les indicateurs testés, comparativement à Nyalenda (périurbain) où les résultats fluctuaient de 50 % à 90 %. Les résultats concernant la fiabilité étaient variables dans les milieux périurbains pour les indicateurs mesurés, alors que les milieux ruraux présentaient des résultats plus stables. Le même phénomène est présent pour les mesures de validité dans les deux milieux.

En conclusion, les résultats montrent que les ASC peuvent recueillir certains types de données communautaires de façon exacte et fiable, ce qui permet de réduire les coûts, particulièrement dans les milieux où les ressources se font rares.

## Background

Reliability and validity of measurements are important for the interpretation and generalisation of research findings [1]. Valid, reliable, and comparable measures of health states of individuals are critical components of the evidence base for health policy [2]. Understanding the validity and accuracy of data is important so that such data can be used with confidence, or at least with knowledge of its limitations. The need for sound information is especially urgent in the case of emergent diseases and other acute health threats, where rapid awareness, investigation and response can save lives and prevent broader national outbreaks and even global pandemics [3].

The government of Kenya, through the Ministry of Public Health and Sanitation has rolled out the community health strategy as a way of improving health care at the household level. This strategy involves CHWs collecting health status data at the household level, which is presented at community meetings in which the community discusses the results, identifies priority actions, and plans activities for improving indicators found to be low, in order to improve their health status.

A lot of successful health interventions in many parts of the developing world have involved the community health workers in reaching out to the community [4]. Large scale involvement of community health workers in government initiatives and most especially to collect health data for use in health systems has been minimal, perhaps due to the assumption that the data may not be reliable enough for decision making in the formal health sector.

Western Kenya has consistently provided low health and development indicators despite an array of interventions initiated by NGOs and the Government of Kenya. These poor indicators beg for concerted efforts to ensure that a reversal of the poor trends is achieved. Future interventions require valid and accurate information on the health status of the population for effective planning, monitoring, and evaluation to track effectiveness. Available information may not always be timely, complete, or relevant to the local context [5].

Population-based sample surveys and sentinel surveillance methods, such as Demographic and Health Surveys, are commonly used as substitutes for routinely collected data. Nevertheless, these methods have been criticised for being expensive, providing inadequate coverage of the population, and lacking in timeliness.

With the rolling out of the Community Health Strategy, community health status information became readily available.

Community health workers (CHWs) and other lay community workers collect a wide range of health information. However, little is known as to whether this information can be relied on to measure population health status, and the causes and distribution of disease. CHWs’ job description included health education and basic preventive services for family planning; maternal and child health; improving nutrition; basic hygiene, sanitation; and child immunization [12].

Today it also includes mass immunization for polio eradication, newborn care, referral of eligible cases to health facilities, and regular record-keeping for updating the community health information system [7,12]. This implies that collection of health information is a role that has been shifted to CHWs in recent times. Results of a study done in Zambia indicated that CHWs can also prepare and interpret malaria rapid diagnostic tests correctly and safely when supported by clear instructions and appropriate training [6].

A study by Kisia and others found that community health workers, with supervision from the facility staff, collect and analyze data, and produce information which was to be used to decide which health problems the community needed to address. The basic objective of data collection by CHWs was to improve their own work, management and output. Through such an arrangement, the community was enabled to address some of its health-related problems with its own resources (for example, construction of latrines) [7]. This demonstrates that in resource poor settings, CHWs can be used to collect data for planning for interventions at the community level.

It is therefore necessary to determine the validity and reliability of the data collected by community health workers, in order to establish its usefulness for planning and policy formulation for the communities from which it is collected. This would go a long way to settle speculation on whether the data collected by these workers is robust enough for use in determining the health and disease distribution in a population [4].

### Objective

The purpose of this study was to determine the validity and reliability of data collected by CHWs in different socio-economic contexts in Kenya.

## Methods

### Description and selection of study sites

Community Units that were implementing the Community Strategy as piloted by the Ministry of Health were purposely included in the study. Of these sites, the socio-economic context of each site was taken into consideration to reflect rural agrarian where the community relies on crop agriculture as a major economic activity, and peri-urban where the community relies on different economic activities. The peri-urban site exhibits a slum-like environment where social amenities are scarce and this is compounded by high population density.

### Data collection

Community health workers registered and updated individual members of households’ information twice a year as required by the Community Strategy using the household register, a tool provided by the Ministry of Health. Special permission was sought to access this data from the community health committee. Ten percent of this data was re-collected using the same tool by a technically trained group of final year community health and development Bachelor of Science students, who were recruited as research assistants for the study, providing the standard for the data collection, in order to validate the data. Systematic random sampling was applied with the list of households being obtained from the CHWs data used as the sample frame. The research assistants visited the selected households and interviewed the same respondents that were interviewed by the CHWs. Where these respondents were unavailable, a call back was made at a time when they would be around. In case of migration, especially in the peri-urban site, the household was replaced with another household by the lead researcher. The first wave of the data collection by the CHWs was conducted in March 2011. The second wave followed at most two weeks later, depending on the site.

This study analyzed the consistency in repeated self-reports of health indicators over two interview waves. A total of 9906 households were visited by CHWs. Of these 4612 were in Butere, the rural site, while 5294 were in Nyalenda, the peri-urban site. Apart from their training in community health and development, the students were also trained in research methods and data collection techniques. The sample size for this study was 1015, which is the total number of households visited by the research assistants, 472 in Butere and 543 in Nyalenda.

The study used the Test-Retest/Stability Reliability which compares results from an initial test with repeated measures later on, the assumption being that if the instrument is reliable there will be close agreement over repeated tests if the variables being measured remain unchanged. The Kappa score, specificity, and positive predictive values (PPVs) were also used to measure reliability and validity, respectively [8]. Table 1 displays the manner in which specificity and predictive value were calculated.

**Table 1:** Two-by-two table for calculation of specificity and predictive values

		Test results (CHW data)	
	
		Positive (+) (yes)	Negative (-) (no)
**Research assistant data**	Positive (+) (yes)	a	b
	
	Negative (-) (no)	c	d

Specificity = d / c+d

Positive predictive value = a / a+c

Kappa measures the difference between observed and expected agreement, and is standardized to lie on a -1 to 1 scale, where 1 is perfect agreement, 0 is exactly what would be expected by chance, and negative values indicate agreement less than chance, i.e., potential systematic disagreement between the observers. This was ranked as follows for this study, as in a study conducted by Rietveld and van Hout in 1993: < 0 Less than chance agreement, 0.01–0.20 Slight agreement, 0.21– 0.40 Fair agreement, 0.41–0.60 Moderate agreement, 0.61–0.80 Substantial agreement, 0.81–0.99 Almost perfect agreement [13].

## Results

The study was conducted in two research sites: a peri-urban informal settlement (Nyalenda), essentially a slum area, with many unplanned structures; and a rural site (Butere). The two sites differed in socio-economic characteristics and the composition of the community health workers recruited. The indicators tested were the measles vaccine, antenatal attendance by mothers four times or more during the last pregnancy with the youngest child under five years, and skilled attendant assisted delivery for the same youngest child under five years. These indicators are relevant to the fourth and fifth millennium development goals tracked by these communities.

### Intra-site comparisons by indicators of reliability and validity of data

#### Reliability measurements for peri-urban site (Nyalenda)

The observed difference for the age variable in Nyalenda was 0.66, portraying very little inter observer difference scores between the two types of data collectors. The gender variable showed slight agreement between the two sets of data. The maternal and child health indicators showed agreements ranging from less than chance to substantial agreement, with less than chance agreement in the measles variable and substantial agreement in health facility delivery. Table 2 gives a summary of the reliability measurements of selected variables in Nyalenda.

**Table 2:** Reliability and validity of demographic, maternal and child health indicators in Nyalenda (peri-urban site)

		Research assistant data	Test results	Difference	Kappa score	Agreement	Specificity	PPV
*Age		19.2	18.6	0.7	**-**	**-**		

**Sex**	***Male*** ***Female***	46.8 53.2	46.1 53.9	0.7 -0.7	0.07	Slight		

**Measles vaccine received**	***Yes*** ***No***	85.5 14.5	75.7 24.3	9.8 -9.8	-0.97	< chance	59.67	88.5

**ANC 4+**	***Yes*** ***No***	65.3 34.7	65.8 34.2	-0.5 **0.5**	0.50	Moderate	98.5	99.2

**Skilled attendant delivery**	***Yes*** ***No***	68.1 31.9	60.5 39.5	7.6 -7.6	0.75	Substantial	80.7	88.8

Age indicated is mean age of household members.

#### Validity measurements for Nyalenda

The maternal and child health indicators ranged from 59.67 to 98.5 for the specificity values and 88.53 to 99.2 for the PPV. Measles registered the lowest specificity values, as indicated in table 2.

#### Reliability measurements for Butere

The observed agreement in the mean age was 23.34 for the research assistant data and 21.69 for the test results in Butere giving an observed difference of 1.65. The Kappa rating ranged from chance to slight agreement, giving low reliability estimates for this site. Table 3 presents the summary of these measures.

**Table 3:** Reliability and validity of demographic, maternal and child health indicators in Butere (rural site)

		Research assistant data	Test results	Difference	Kappa score	Agreement	Specificity	PPV
*Age		23.34	21.69	1.65	**-**	**-**		

**Sex**	***Male*** ***Female***	50.6 49.4	48.6 51.4	2.00 -2.00	0.12	Slight		

**Measles vaccine received**	***Yes*** ***No***	77.8 22.2	79.5 20.5	-1.7 1.7	0.16	Slight	**92.3**	**97.9**

**ANC 4+**	***Yes*** ***No***	57.1 42.9	57.2 42.8	-0.1 0.1	0.76	Substantial	**99.7**	**99.8**

**Skilled attendant delivery**	***Yes*** ***No***	30.2 69.8	31.1 68.9	-0.9 0.9	0.04	Slight	**98.7**	**97.1**

Age indicated is mean age of household members.

#### Validity measurements for Butere

Specificity and positive predictive values for the indicators in Butere ranged from 92.34 to 99.7 and 97.1 to 99.8, respectively. Generally, validity estimates for these indicators were very high, as shown in table 3.

### Inter-site comparison of reliability and validity measures

Nyalenda had better agreement in all variables as compared to Butere. The Nyalenda scores spread out from less than chance agreement to moderate agreement while Butere scores clustered together at slight agreement. Generally, Butere presented better results than Nyalenda across the board. Nyalenda showed lower specificity for the immunization variable. Butere results presented better specificity measures as well as positive predictive value, while Nyalenda had an outlier (measles) in the specificity measures.

## Discussion

### Reliability of data collected at the community level by community health workers

This study analyzed the consistency in repeated self-reports of health indicators over two interview waves. Overall, there was a high level of agreement between the research assistant data and the test results. This suggests that the use of CHWs provides a reliable method for collecting data especially on maternal health indicators. The reliability of the measles vaccine which gave a Kappa statistic rating of less than chance in Nyalenda also had the highest inter-observer difference among all the variables. This may be due to the fact that inasmuch as the measles vaccine is administered at a particular time in the child’s life, the vaccine had been administered to all children under five years of age due to an outbreak in the region during the study, and not necessarily according to the schedule.

Therefore, recall may have been clouded or confusing for the respondent. This may have caused the variance between the observers. The remaining variables showed ratings of between chance to substantial agreements between the research assistant data and the test data. Viera and colleagues [8] observed that with a large enough sample size, 1000 and above, any Kappa score above 0 will become statistically significant, and that it is not important if one observer differs from another slightly, as long as the diagnosis is positive or negative for both, and not positive for one observer and negative for the other. In a study conducted in the United States, Li *et al.* also found that there was consistency in the estimates of key health indicators when the national Behavioral Risk Factor Surveillance System data was compared to particular National health surveys [14]. The relative differences between the two were found to be ranging from 0.2% to 17.1%. This indicates the findings are not only unique to Kenya but shared with other developing countries. However, differences were noted in the methods; the US study was more surveillance than updating of a register. Also, it was more rigorously done since it included a research component. Another study conducted at the University of Illinois by Hayes and Nardulli, 2011 [15] found that coders are able to extract precise information at least 84% of the time, with the average coder extracting precise information almost 90% of the time. These results are maintained after coders have completed training and are subjected to blind testing.

The paradox presented by the measles and antenatal clinic (ANC) variables where the percentage difference between the two data sets (research assistant data and test data) were low (high agreement between the two observers) but the coefficient of Kappa was unexpectedly low can be explained as in [9,10] high agreement but low Kappa. This paradox extends the assumption that each observer had a relatively fixed prior probability of making positive or negative responses. Influencers of reliability also vary between collectors due to their prior background and experiences. As shown by Hayes and Nardulli, training is one of the factors that can influence reliability and maintenance of reliability in further data collection activities.

### Validity of data collected at the community level by community health workers

Reliability is a necessary, but not sufficient, component of validity. An instrument that does not yield reliable scores does not permit valid interpretations. Validity in this study was estimated using specificity, the probability of true negatives, and positive predictive values, which reflected the probability that an observation as classified from self-reported information was truly as observed.

PPVs also varied between the sites with Butere having higher values than Nyalenda, but decreased slightly in the measles and skilled delivery variables. Sensitivity for detecting true positives in our study, as in other studies [11], was very high, typically between 88.5% and 99.8%. This indicated that few individuals reported values that disagreed while examining the presence of a particular variable. Specificity on the other hand was more variable, with values ranging from 59.67% to 99.7%. Previous research has been consistent in finding PPVs that were higher in urban than in rural settings. This was not the case in our study, where PPV for rural ranged from 99.8% to 88.53% and from 99.2% to 88.5% in peri-urban indicating no significant variations between the two sites.

### Study limitations

The data by community health workers was a complete census of the households by a large number of observers while the validation data was a 10% sample, collected by a small team of research assistants. It is possible that comparison of the two data sets was influenced by the huge differences in sample sizes as well as the number of observers, over and above the quality of data that was being measured. The study results may not be generalizable to other parts for the country due to contextual, social or cultural settings experienced by different communities. Factors affecting reliability and validity, such as heterogeneity of the group being studied, age, educational background, etc., may vary due to inherent differences in communities, and therefore CHWs.

## Conclusions

Results suggest that validity, as measured, does not vary significantly when the two sites are compared. The differences noted in the specificity ratings between the sites, especially in the immunization variable, are also consistent with the reliability measures within the same variable in the analysis.

Although there was some variability in the measurements recorded by the CHWs and the research assistants, there is substantial agreement in maternal health data from both sources. This means that trained CHWs from communities can collect reliable data, especially on maternal and child health indicators. They are therefore a reliable, alternative source of data collection for community based studies. This data can therefore be used for planning and action at the source of collection and at higher levels, for example at the district level.

Future research can be undertaken to establish factors that influence reliability and validity of such kinds of data. This would provide insight on the reasons for differences in the measures between the sites.

## List of abbreviations

ANC: Antenatal clinic; CHWs: Community health workers; NGO: Non-governmental organization; PPVs: Positive predictive values

## Competing interests

The authors declare no competing interests.

## Authors' contributions

Both authors contributed equally in writing this article.

## Declarations

The publication costs associated with this article are funded by Foreign Affairs, Trade and Development Canada and the International Development Research Centre through the Global Health Research Initiative.

This article has been published as part of *BMC Health Services Research* Volume 14 Supplement 1, 2014: Uptake and impact of research for evidence-based practice: lessons from the Africa Health Systems Initiative's research component. The full contents of the supplement are available online at http://www.biomedcentral.com/bmchealthservres/supplements/14/S1
